# Ethnopharmacological survey of medicinal plants practiced by traditional healers and herbalists for treatment of some urological diseases in the West Bank/Palestine

**DOI:** 10.1186/s12906-017-1758-4

**Published:** 2017-05-08

**Authors:** Nidal Amin Jaradat, Abdel Naser Zaid, Rowa Al-Ramahi, Malik A. Alqub, Fatima Hussein, Zakaria Hamdan, Mahmoud Mustafa, Mohammad Qneibi, Iyad Ali

**Affiliations:** 1 Department of Pharmacy, Faculty of Medicine and Health Sciences, An-Najah National University, Nablus, P.O. Box 7 Palestine; 2 Department of Biomedical Sciences, Faculty of Medicine and Health Sciences, An-Najah National University, Nablus, P.O. Box 7 Palestine; 3 Nephrology Unit, Internal Medicine Department, An-Najah National University Hospital, Nablus, Palestine; 4 Department of Urology, Faculty of Medicine and Health Sciences, An-Najah University Hospital, An-Najah National University, Nablus, P.O. Box 7 Palestine

**Keywords:** Ethnopharmacology, Urological diseases, Herbalists, Traditional healers, Palestine

## Abstract

**Background:**

Throughout history, every civilization in the world used plants or their derivatives for treatment or prevention of diseases. In Palestine as in many other countries, herbal medicines are broadly used in the treatment of wide range of diseases including urological diseases. The main objective of this research is to study the use of herbal remedies by herbalists and traditional healers for treatment of various urological diseases in the West Bank regions of Palestine and to assess their efficacy and safety through the literature review of the most cited plants.

**Method:**

The study included a survey part, plant identification and a review study. The first part was a cross-sectional descriptive study. Face to face questionnaires were distributed to 150 traditional healers and herbalist in all regions of the West Bank of Palestine. The literature review part was to assess the most cited plants for their efficacy and toxicity.

**Results:**

One hundred forty four herbalists and traditional healers accepted to participate in this study which was conducted between March and April, 2016. The results showed that 57 plant species belonging to 30 families were used by herbalists and traditional healers for treatment of various urinary tract diseases in Palestine. Of these, Apiaceae family was the most prevalent. *Paronychia argentea*, *Plantago ovata*, *Punica granatum*, *Taraxacum syriacum*, *Morus alba* and *Foeniculum vulgare* were the most commonly used plant species in the treatment of kidney stones, while *Capsella bursa-pastoris*, *Ammi visnaga* and *Ammi majus* were the most recommended species for treatment of urinary tract infections and *Portulaca oleracea* used for renal failure. In addition *Curcuma longa* and *Crocus sativus* were used for enuresis while *Juglans regia*, *Quercus infectoria*, *Sambucus ebulus* and *Zea mays* were used for treatment symptoms of benign prostate hyperplasia. Fruits were the most common parts used, and a decoction was the most commonly used method of preparation. Through literature review, it was found that *Paronychia argentea* has a low hemolytic effect and contains oxalic acid and nitrate. Therefore, it could be harmful to renal failure patients, also *Juglans regia*, *Quercus infectoria* and, *Sambucus ebulus* are harmful plants and cannot be used for treatment of any disease.

**Conclusions:**

Our data provided that ethnopharmacological flora in the West Bank regions of Palestine can be quite wealthy and diverse in the treatments of urinary tract diseases. Clinical trials and pharmacological tests are required evaluate safety and efficacy of these herbal remedies.

## Background

Traditional herbal medicine is an important part of all nations’ history, therefore, an establishment of the original uses and local names of plants has significant potential societal benefits. Unfortunately, recently with the fast growth in the technical aspects of the world, loss of customs and various ethnic cultures, some of this information may disappear [1–3]. Since the beginning of history, human beings have used plants as medicine, and Ancient Arabic Medicine was influenced by medicinal practices in India, Persia, Mesopotamia, Spain, Rome and Greece [4].

Palestine had high ecosystem diversity due to its geographical location between Africa, Asia, and Europe and due to different climatic, zoogeographic, and phytogeographic zones, this creates great biological diversity [5, 6]. In the West Bank regions of Palestine, traditional medicine is widely used especially in rural areas; this may be due to the political conflicts in this country and the cost of conventional drugs [7–9]. Hundreds of shrubs, trees, and herbal species used as antipyretics, analgesics, diuretics, laxatives, antimicrobial, antidiarrheal, emetics and cardio-tonics in the West Bank area of Palestine [10]. These plants are available and cheap because they grow wildly in nature or cultivated [11, 12].

The rich variety of approaches employed by herbalists and traditional healers to treat disorders and diseases of the urinary tract is indicative of the depth and breadth of indigenous medicine practiced among these traditional healers and herbalists in the twentieth century [12].

The Kidney is the organ that has numerous physiological functions. Its role is to maintain the homeostatic balance of body fluids and electrolytes. Kidneys are vital regulators of glucose metabolism, blood pressure, and erythropoiesis. Patients with kidney diseases have significant morbidity and mortality [13, 14].

There are many urologic diseases and disorders. According to the American Urological Association Foundation, the most commonly identified urological diseases include hyperplasia, benign prostate hyperplasia (BPH), urinary tract infections, urethral and kidney stones, enuresis (urinary incontinence) and renal failure [15].

According to the Palestinian Central Bureau of Statistics and Ministry of Health annual report in 2014, the visits to the outpatient urological clinics of governmental hospitals in Palestine were 49,275 visits per year. Moreover, about 4% of death causes in Palestine were due to renal failure and other kidney diseases. In the USA, about 26 million American adults have kidney disease, and it is considered the 9th leading cause of death in the United States. Kidney diseases kill more people than breast or prostate cancer yearly [16–18].

For these reasons, this study aimed to collect data from herbalists and traditional healers about the folk herbal remedies, which have been utilized for treatment of urological diseases in the West Bank regions of Palestine and to verify their pharmacological and toxicological effects through literature review.

## Methods

### Study areas

West Bank is an important territory of Palestine. The climate in the West Bank is mostly Mediterranean, slightly colder in mountains and hills compared with the shorelines in the western lands. In the east, it includes the desert and the shoreline of the Dead Sea, both with dry and hot climate. The shores of the Dead Sea are about 430 m below sea level, and it is considered the Earth’s lowest elevation on the land. Accordingly, all these factors explain the enormous diversity of the West Bank flora. This diversity is directly reflected in the distribution and diversification of agricultural patterns, from the rain-fed farming in the mountains (Jerusalem, Ramallah, Hebron, Nablus, Bethlehem, Salfeit) to an irrigated agriculture as in Jenin, Tobas, Toulkarem, Qalqilya and Jericho lands [19, 20].

### Selection of informants

**Fig. 1:**
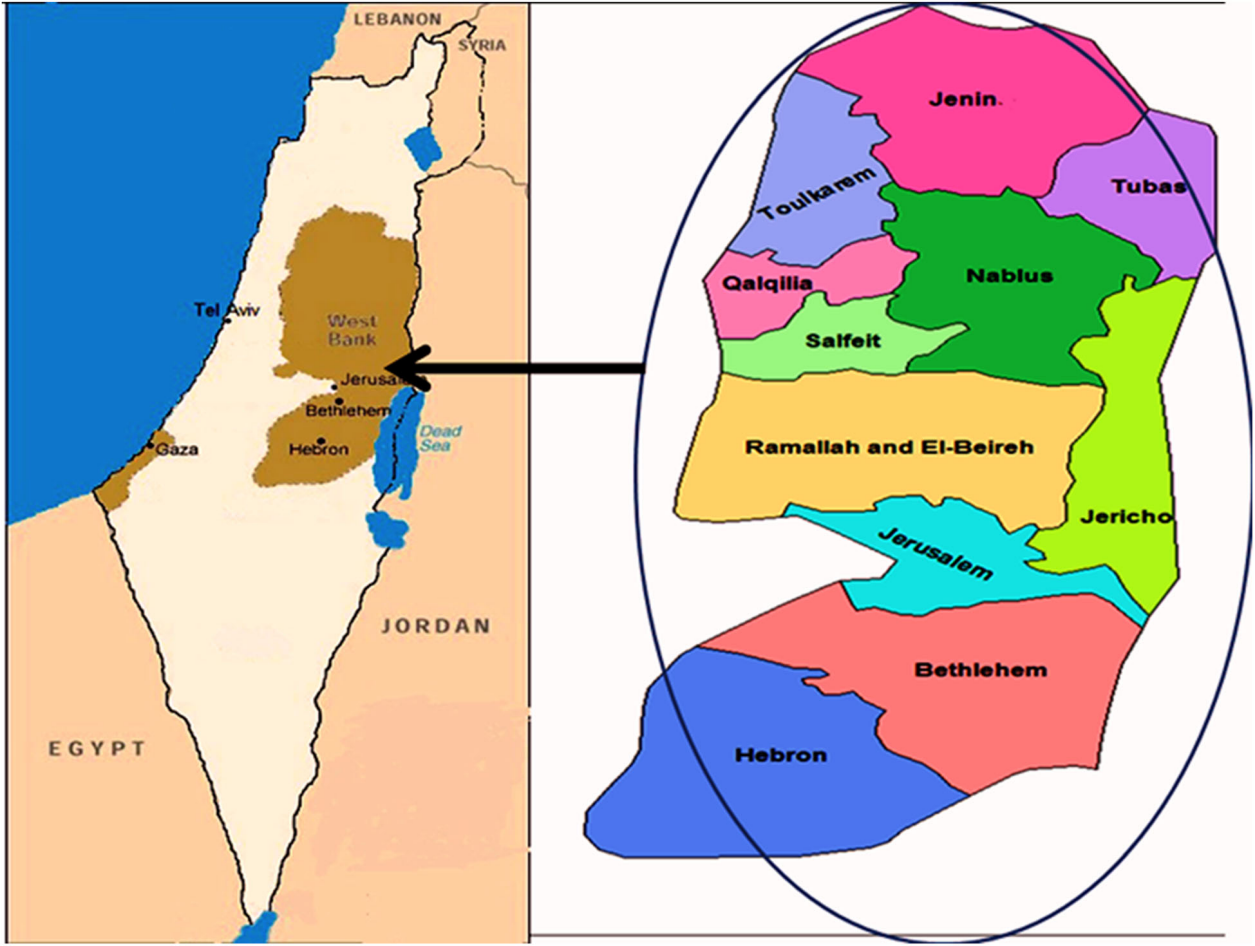
The study area map showing all the surveyed regions in the West Bank/Palestine

An ethnopharmacological survey (questionnaire-based cross-sectional descriptive study) was used. Areas visited included all regions of the West Bank/Palestine, including Nablus, Jenin, Tubas, Toulkarem, Salfeit, Qalqilya, Ramallah, Jericho, Jerusalem, Bethlehem and Hebron (Fig. 1) between March and April 2016. The Institutional Review Board (IRB) at An-Najah National University approved the study protocol and the informed consent forms (IRB number was 134/February/2016). The study was conducted in accordance with the requirements of the declarations of Helsinki (World Medical Association 2008), the current Good Clinical Practice (GCP) Guidelines (EME 1997) and the International Conference on Harmonization (ICH1996) Guidelines, and a written informed consent was obtained from the participants.

The objectives of the study were explained to the participants, they were not offered any incentives, and they were able to withdraw from this study at any time.

A total of 150 traditional healers and herbalists were interviewed in this study. 144 participants accepted to answer the questionnaire (102 males and 42 females), where 26 of them were from Hebron (16 males and 10 females), 18 from Jenin (12 males and 6 females), 17 from Jericho (14 males and 3 females), 16 Nablus and Qalqilya (12 males and 4 females from Nablus) (10 males and 6 females from Qalqilya), 14 from Toulkarem (12 males and 2 females), 11 from Ramallah (9 males and 2 females), 9 from Jerusalem (7 males and 2 females), 6 from Tubas and Jerusalem (4 males and 2 females from Tubas) and (3 males and 3 females from Jerusalem), and 5 from Salfeit (3 males and 2 females), from 11 regions of the West Bank Area of Palestine. They were from different ages (22–91 years) and were selected with the help of local people. The selected healers were well known in the community due to their long practice of providing services related to traditional health care. All the participants (i.e., 144) were asked to provide information on the plant(s) that they use for treating the urinary tract diseases, parts of the plants used such as leaves, roots, flowers, stems and seeds, methods of preparation (e.g decoction, juice, infusion, powder), and methods of administration (either orally or topically). In addition to their opinion about the advantages of herbal remedies.

The study was a face to face questionnaire. This method has proven to be a very practical and useful option of data collection. The survey was anonymous, pretested by a pilot study for reliability, validity and clarity of the questionnaire. Meanwhile, the duration of the interviews ranged from 20 to 60 min, with one visit per interviewee in each case. Interviews were conducted in Arabic, the local language of the informants and the plant names were given in Arabic and later translated into English and Latin using reference books as well as about 50% of these Arabic names were linked to an actual right scientific name [21–23].

### Plant identification

The collected plant samples from these informants were stored in the pharmacognosy laboratory at the Pharmacy Department, An-Najah National University in appropriate glassware and individual herbarium wooden frames. They were identified later by a team of teaching assistants and technicians under the supervision of the pharmacognosist (Dr. Nidal Jaradat), all fifty-seven plants’ species that were mentioned by informants, were identified by using photographs from reference books and dried herbarium specimens [24, 25].

### Data analysis

The frequency of citation (FC) for all plants species in this study were calculated by using the following formula:

FC = (Number of times a particular species was mentioned by herbalists and traditional healers/a total number of occasions that all species were mentioned) × 100 [24].

To evaluate the relative importance of plants in indigenous healthcare systems, the use value (UV) is used as a micro-statistical tool, which reflects people interaction with specific plants as the best treatments for urinary tract diseases. It is a quantitative method that can be used to prove the relative importance of species known locally. It can be calculated according to the following equation:

$$ \mathrm{UV}=\frac{\sum U}{n} $$

Where UV is the use value of a species; *U* is the number of citations per species; *n* is the number of informants [25].

Factor of informant’s consensus (F_ic_) was calculated according to the following equation:

$$ {F}_{ic}=\frac{Nur- Nt}{Nur-1} $$

Where Nur is the number of use citations in urinary tract disease category, and Nt is the number of taxa used for the treatment of these diseases.

This factor is employed to indicate how homogenous the information is. F_ic_ value is close to 0 if plants are chosen randomly, or if informants do not exchange information about their use. High values of Fic (close to 1) occur when there is a clear selection criterion in the community and if information is frequently exchanged between informants [26].

The Choice Value (CV) method is a valuable assessment tool to measure related plant species for treatment of urinary tract diseases [27].

The CV is calculated as in the following equation:

$$ CV\kern0.5em  species=\frac{Pcs}{Sc}\times 100 $$

*Pcs*: percent of informants that cited certain plant species for the treatment of urinary tract disease.

*Sc*: is the total number of species mentioned for treatment of disease by all informants. Choice values are ranked from 0 to 100 with 100 indicating complete preference and fewer alternatives.

The significance of medicinal plant families was assessed using the Family Use Values (FUV), which was calculated according to the following equation:

$$ FUV=\frac{UVs}{(ns)} $$

where, *UVs* = use values of the taxa, and *ns* = total number of species within each family which were used for the treatment of urological diseases in the West Bank area of Palestine [28].

### Review study

A literature review was conducted by a systematic search of the scientific literature, which was published before January 2017, by using Medline, Pubmed, Scopus and Google Scholar electronic searching machines. It cited most of the plants which had FC higher than 50% and their applications in the treatment of urological disease. The investigators practiced the following Keywords: folk uses for urinary tract, ethnopharmacological uses urinary tract, traditional methods of urinary tract, evidence-based uses, toxicities and side effects for individual plant names.

## Results

### Socio-demographic factors

As shown in Table 1, most of the respondents who worked in this field were males. Most of them had high educational levels. In fact, 30.56% of the interviewed were secondary school graduates. The majority of respondents were from areas of the West Bank that mostly depended on agriculture or grazing as a mean of income (Hebron, Jenin, and Jericho).

**Table 1:** Sociodemographic factors related to the respondents

Variable	Number of herbalists and traditional healers (*N* = 144)
Gender	
Male	102
Female	42
Education level	
No formal education	32
Elementary	14
Secondary school	28
High secondary school	44
Undergraduate	22
Graduate (higher education)	4
Residency	
Bethlehem	6
Hebron	26
Jenin	18
Jericho	17
Jerusalem	9
Nablus	16
Qalqilya	16
Ramallah	11
Salfeit	5
Tubas	6
Toulkarem	14
Age (mean ± SD) years	54.8 ± 17.9
Experience (mean ± SD) years	29.1 ± 10.9

Regarding training and knowledge acquisition; (i) 77% of the respondents acquired their skills through observing their family members, (ii) 21% gained their skills through coursework and apprenticeship, (iii) and about 2% claimed they had a divine gift for the healing of certain diseases, which means that most of them had this knowledge through their families’ historical healing knowledge skill.

Data collection of ethnopharmacological plants.

The fruits were the most commonly used parts of plants for the treatment of urinary tract diseases followed by seeds and roots. The modes and methods of preparation varied considerably from one healer to another; however, all of these methods were administered orally as described by the interviewees and shown in Table 2.

**Table 2:** The medicinal plants used for the treatment of some urinary tract diseases, the plant parts used, use values, choice value, frequency of citation, modes and methods of preparation

Scientific names	Arabic local names	English common names	Family	Voucher specimen codes	Urinary tract diseases	Part used and mode of preparation	Method of preparation	# Informant 144	FC, %	UV	CV
*Paronychia argentea* Lam.	رجل الحمام	Chickweed, Algerian tea	Caryophyllaceae	Pharm-PCT-1793	Kidney stones	Entire plant/Boil about 100 g of the plant in 100 ml water. 30 ml of this decoction is to be given orally before each meal.	Decoction	140	97.22	0.97	1.71
*Curcuma longa* L.	كركم	Turmeric	Zingiberaceae	Pharm-PCT-2709	Enuresis	Roots/Steep 150 g of the roots in 200 ml water for two hours. This infusion is to be given after each meal.	Infusion	135	93.75	0.94	1.64
*Plantago ovata* Forssk.	لسان الحمل البيضوي	Blond Psyllium	Plantaginaceae	Pharm-PCT-1891	Kidney stones	Seeds/About 50 g of dried and ground seeds are to be given orally once daily.	Powder	134	93.06	0.93	1.63
*Portulaca oleracea* L.	فرفحينه	Common Purslane	Portulacaceae	Pharm-PCT-1935	Renal failure	Entire plant/10 drops of the fresh plant juice are to be given twice daily.	Juice	134	93.06	0.93	1.63
*Punica granatum* L.	رمان	Pomegranate	Lythraceae	Pharm-PCT-2721	Kidney stones	Fruits/300 ml of Pomegranate juice obtained from the fruits is to be given five times a day.	Juice	133	92.36	0.92	1.62
*Juglans regia* L.	جوز	Walnuts	Juglandaceae	Pharm-PCT-2714	Prostatic enlargement	Bark/Boil 100 g from the ground bark in 300 ml water for 30 min. 50 ml of this decoction is to be given twice daily.	Decoction	132	91.67	0.92	1.61
*Capsella bursa-pastoris* (L.) Medik.	کیس الراعي	Shepherd’s-Purse	Brassicaceae	Pharm-PCT-497	Urinary tract infection	Fruits/ Boil about 100 g from the ground fruits in 300 ml water. Give 100 ml three times a day after meals.	Decoction	132	91.67	0.92	1.61
*Quercus infectoria subsp. veneris* (A.Kern.) Meikle	بلوطحلبي	Aleppo Oak	Fagaceae	Pharm-PCT-1977	Prostatic enlargement	Entire plant/Boil about 50 g of the any plant part in 200 ml water. 50 ml of this decoction is to be given four times a day with meals.	Decoction	119	82.64	0.83	1.45
*Crocus sativus* L.	زعفران	Saffron	Iridaceae	Pharm-PCT-2733	Enuresis	Flowers/Steep 40 g of the flowers in 100 ml boiled water for 8 h. 15 ml from this infusion is to be given two times a day	Infusion	118	81.94	0.82	1.44
*Hibiscus sabdariffa* L.	كركديه	Roselle	Malvaceae	Pharm-PCT-2752	Urinary tract infection	Flower/Boil about 150 g of the plant in 100 ml water. 40 ml of this decoction is to be given orally before each meal.	Decoction	112	77.78	0.78	1.36
*Ammi visnaga* (L.) Lam.	الخله المصريه	Khella	Apiaceae	Pharm-PCT-139	Urinary tract infection	Fruits/Boil about 250 g of the plant in 500 ml water. 100 ml of this decoction is to be given orally three to five times a day.	Decoction	102	70.83	0.71	1.24
*Ammi majus* L.	الخله الشيطانيه	Bishop’s Flower, Bishop’s weed	Apiaceae	Pharm-PCT-138	Urinary tract infection	Fruits/Boil about 30 g of fruits in 100 ml water for 5 min. This decoction is to be given three times a day after meals.	Decoction	98	68.06	0.68	1.19
*Taraxacum syriacum* Boiss.	هندباء	Silkweed	Compositae	Pharm-PCT-2396	Kidney stones	Roots/Boil about 300 g of the plant in 500 ml water. 100 ml of this decoction is to be given orally before each meal.	Decoction	95	65.97	0.66	1.16
*Sambucus ebulus* L*.*	البيلسان	Danewort	Adoxaceae	Pharm-PCT-2135	Enuresis, prostatic enlargement.	Leaves/Steep 200 g of the powdered leaves in 500 ml boiled water for 12 h. About 100 ml from this infusion is to be given three times daily.	Infusion	92	63.89	0.64	1.12
*Morus alba* L.	توت ابيض	White Mulberry	Moraceae	Pharm-PCT-2750	Kidney stones	Fruits/150 ml of fresh juice are to be given orally every two hours.	Juice	91	63.19	0.63	1.11
*Zea mays* L.	ذره	Maize	Poaceae	Pharm-PCT-2747	Prostatic enlargement, urinary tract infections	Flower stigmas (corn silk)	Infusion	72	50.00	0.58	1.02
*Foeniculum vulgare* Mill.	شومر	Fennel	Apiaceae	Pharm-PCT-1041	Kidney stones, urinary tract infection	Fruits/ Boil about 50 g of the fruits in 50 ml water. 25 ml of this decoction is to be given three times a day.	Decoction	78	54.17	0.54	0.95
*Daucus carota* L.	جزر بري	Wild carrot	Apiaceae	Pharm-PCT-829	Urinary tract infection	Fruits/ Boil about 150 g of the powder in 600 ml water. About 150 ml of this decoction is to be given three times a day.	Decoction	69	47.92	0.48	0.84
*Buddleja coriacea* Remy	عفار	Butterfly bush	Scrophulariaceae	Pharm-PCT-2746	Enuresis	Flowers/Boil about 20 g of the flowers in 200 ml water. About 50 ml of this decoction is to be given once daily.	Infusion	67	46.53	0.47	0.82
*Persea americana* Mill.	افوكادو	Avocado	Lauraceae	Pharm-PCT-2740	Enuresis	Leaves/Boil about 50 g of leaves in 150 ml water for 5 min. This decoction is to be given three times a day after meals.	Decoction	66	45.83	0.46	0.80
*Cucurbita pepo* L.	قرع	Pumpkin	Cucurbitaceae	Pharm-PCT-2762	Prostatic enlargement	Seeds/ Boil about 100 g of the seeds in 600 ml water. About 50 ml of this decoction is to be given three times a day.	Decoction	66	45.83	0.46	0.80
*Equisetum ramosissimum* Desf.	كنباث أفرع	Branched Horsetail	Equisetaceae	Pharm-PCT-915	Renal failure	Entire plant/ Boil about 50 g of the powder in 600 ml water. About 100 ml of this decoction is to be given three times a day.	Decoction	66	45.83	0.46	0.80
*Ferula communis* L.	كلخ	Common Giant Fennel	Apiaceae	Pharm-PCT-1016	Kidney stones	Fruits/Steep 50 g of the plant in 150 ml boiled water for 12 h. 10 ml from this infusion is to be given two times a day	Infusion	65	45.14	0.45	0.79
*Rosa canina* L.	ورد السياج	Rose hips, Dog-rose	Rosaceae	Pharm-PCT-2052	Urinary tract infection	Fruits/Boil about 70 g of leaves in 150 ml water for 15 min. This decoction is to be given three times a day after meals.	Decoction	65	45.14	0.45	0.79
*Ficus sycomorus* L.	جميز	Fig-Mulberry	Moraceae	Pharm-PCT-1030	Urinary tract infection, renal failure	Fruits/Extract 100 ml of juice to be given orally five times a day.	Juice	63	43.75	0.44	0.77
*Petroselinum crispum* (Mill.) Fuss	بقدونس	Garden Parsley	Apiaceae	Pharm-PCT-2739	Prostatic enlargement, urinary tract infections, kidney stones	Fruits/Boil about 100 g from the powdered fruits in 600 ml water. About 150 ml from this decoction is to be given 4–5 times daily.	Decoction	44	30.56	0.43	0.76
*Acacia senegal* (L.) Willd.	الصمغ العربي	Arabic Gum	Leguminosae	Pharm-PCT-2755	Kidney stones	Gum/About 20 g of gum are to be given orally twice daily with a cup of milk.	Powder	61	42.36	0.42	0.74
*Raphanus raphanistrum* L.	فجل بري	Radish	Brassicaceae	Pharm-PCT-2007	Kidney stones	Seeds/ Boil about 250 g of the powder in 750 ml water. About 150 ml of this decoction is to be given three times a day.	Decoction	59	40.97	0.41	0.72
*Olea europaea* L.	زيتون	Olive	Oleaceae	Pharm-PCT-1664	Renal failure	Fruits/Three table spoons of Olive oil is to be given orally twice a day.	Oil	57	39.58	0.40	0.69
*Apium graveolens* L.	كرفس	Celery	Apiaceae	Pharm-PCT-204	Prostatic enlargement	Entire plant/Ten drops of Celery entire plant juice are to be given orally three times a day.	Juice	56	38.89	0.39	0.68
*Astracantha gummifera* (Labill.) Podlech	الكثيراء	Tragacanth	Leguminosae	Pharm-PCT-2754	Kidney stones	Gum/About 50 g of gum are to be given orally three times daily with 2 cups of water.	Powder	55	38.19	0.38	0.67
*Urtica pilulifera* L.	القريص الشائك	Roman Nettle	Urticaceae	Pharm-PCT-2561	Kidney stones, Urinary tract infections	Roots/Boil about 20 g of the powder in 100 ml water. About 30 ml of this decoction is to be given three times a day.	Decoction	55	38.19	0.38	0.67
*Arctostaphylos uva-ursi* (L.) Spreng.	عنب الدب	Bearberry	Ericaceae	Pharm-PCT-2705	Urinary tract infection	Leaves/Boil about 90 g from the dried leaves in 400 ml water. About 50 ml from this decoction is to be given 3–5 times daily.	Decoction	55	38.19	0.38	0.67
*Cinnamomum verum* J.Presl	قرفه	Cinnamon	Lauraceae	Pharm-PCT-2707	Prostatic enlargement	Bark/ Boil about 80 g from the dried powdered plant in 400 ml water. About 50 ml from this decoction is to be given 3–5 times daily.	Decoction	54	37.50	0.38	0.66
*Angelica archangelica* L.	حشيشة الملاك	Wild Celery	Apiaceae	Pharm-PCT-2758	Kidney stones	Roots/Boil about 100 g from the dried powdered plant in 500 ml water. About 50 ml from this decoction is to be given 4–5 times daily.	Decoction	51	35.42	0.35	0.62
*Lolium temulentum* L.	زوان مسكر	Darnel	Poaceae	Pharm-PCT-1453	Enuresis	Seeds/Boil about 200 g from the dried powdered plant in 500 ml water. About 100 ml from this decoction is to be given twice daily.	Decoction	44	30.56	0.31	0.54
*Withania somnifera* (L.) Dunal	سموه	Ashwagandha	Solanaceae	Pharm-PCT-2678	Kidney stones	Roots/Boil about 50 g of the powder in 100 ml water. About 50 ml of this decoction is to be given three times a day after meals.	Decoction	43	29.86	0.30	0.52
*Origanum jordanicum* Danin & Kunne	زعتر	Thyme	Lamiaceae	Pharm-PCT-1729	Kidney stones	Leaves/Boil about 60 g of leaves in 200 ml water for 5 min. This decoction is to be given three times a day after meals.	Decoction	42	29.17	0.29	0.51
*Barbarea vulgaris* R.Br.	جرجير بري	Rocketcress	Brassicaceae	Pharm-PCT-2757	Urinary tract infection	Leaves/Steep 40 g of the powdered leaves in 100 ml water for12 hours. About 30 ml from this infusion is to be given three times daily.	Infusion	42	29.17	0.29	0.51
*Jateorhiza palmata* (Lam.) Miers	ساق الحمام	Calumba	Menispermaceae	Pharm-PCT-2753	Urinary infections	Flowers/Steep 200 g of the powdered flowers in 800 ml water for 8 h. About 50 ml from this infusion is to be given three times daily.	Infusion	40	27.78	0.28	0.49
*Viola kitaibeliana* Schult.	بنفسج	Dwarf Pansy	Violaceae	Pharm-PCT-2656	Kidney stones	Seeds/About 10 drops of seeds oil is to be given orally twice daily.	Oil	35	24.31	0.24	0.43
*Glycyrrhiza glabra* L.	عرق السوس	Licorice	Leguminosae	Pharm-PCT-1128	Enuresis	Roots/Steep 50 g of the powdered roots in 300 ml water for 12 h. About 100 ml from this infusion is to be given four times daily.	Infusion	34	23.61	0.24	0.41
*Ocimum basilicum* L.	ريحان	Basil	Lamiaceae	Pharm-PCT-2717	Kidney stones	Leaves/Steep100 grams of the powdered leaves in 400 ml water for 6 h. About 50 ml from this infusion is to be given five times daily.	Infusion	32	22.22	0.22	0.39
*Hypericum perforatum* L.	سانت جون	St John’s Wort	Hypericaceae	Pharm-PCT-2734	Kidney stones	Flowers Boil about 30 g of plant in 100 ml water for 25 min. This decoction is to be given three times a day after meals.	Decoction	32	22.22	0.22	0.39
*Fragaria vesca* L.	فراوله	Strawberry	Rosaceae	Pharm-PCT-2763	Urinary tract infection	Fruits/ Extract 150 ml of juice to be given orally four times a day.	Juice	29	20.14	0.20	0.35
*Cyperus longus* L.	بردي	Sweet Cyperus	Cyperaceae	Pharm-PCT-808	Renal failure	Entire plant/ Boil about 10 g from the dried powdered plant in 100 ml water. About 20 ml from this decoction is to be given 5–6 times daily.	Decoction	22	15.28	0.15	0.27
*Citrullus colocynthis* (L.) Schrad.	حنظل	Bitter apple	Cucurbitaceae	Pharm-PCT-628	Kidney stones	Seed/ Steep 40 g of the grounded seeds in 100 ml water for 12 h. 25 ml from this infusion is to be given three times a day	Infusion	21	14.58	0.15	0.26
*Brassica nigra* (L.) K.Koch	خردل اسود	Black mustard	Brassicaceae	Pharm-PCT-408	Kidney stones	Seeds/ Steep 50 g of the powder in 300 ml water for four hours. 100 ml of this infusion is to be given 4–5 times a day.	Infusion	18	12.50	0.13	0.22
*Helichrysum sanguineum* (L.) Kostel.	دم المسيح	Red everlasting	Compositae	Pharm-PCT-1170	Renal failure	Entire plant/ Steep 100 g of the powdered plant in 100 ml water for 8 h. About 30 ml from this infusion is to be given three times a day.	Infusion	17	11.81	0.12	0.21
*Lupinus angustifolius* L.	الترمس ضيق الأوراق	Blue lupin	Leguminosae	Pharm-PCT-1477	Urinary tract infection	Roots/ Boil 50 g of the roots in 500 ml water for 30 min. 100 ml of this decoction is to be given twice a day.	Decoction	16	11.11	0.11	0.19
*Phaseolus vulgaris* L.	فاصولياء	Common Bean	Leguminosae	Pharm-PCT-2748	Renal failure	Seeds/ Boil about 40 g of the seeds in 500 ml water. 100 ml of this decoction is to be given three times a day with meals.	Decoction	13	9.03	0.09	0.16
*Vitex agnus-castus* L.	كف مريم	Chaste Tree	Lamiaceae	Pharm-PCT-2663	Renal failure	Fruits/ Extract 200 ml of juice to be given orally five times a day.	Juice	13	9.03	0.09	0.16
*Prunus avium* (L.) L.	كرز حلو	Wild cherry	Rosaceae	Pharm-PCT-2751	Kidney stones	Fruits/ About 100 ml of fresh Wild Cherry juice are to be given orally four times a day.	Juice	8	5.56	0.06	0.10
*Phragmites australis* (Cav.) Trin. ex Steud.	قصب بري	Common Reed	Poaceae	Pharm-PCT-1843	Renal failure	Roots/ Boil about 200 g of the roots in 500 ml water. 100 ml of this decoction is to be given four times a day.	Decoction	8	5.56	0.06	0.10
*Malvella sherardiana* (L.) Jaub. & Spach	خبيزة بريه	Field Mallow	Malvaceae	Pharm-PCT-1508	Renal failure	Leaves/ Steep 20 g of the powdered leaves in 150 ml water for 2–3 h. About 50 ml from this infusion is to be given three times daily.	Infusion	7	4.86	0.05	0.09
*Capsicum annuum* L.	الفليفلة الشجيرية	Chili pepper	Solanaceae	Pharm-PCT-2729	Urinary tract infections	Fruits/ About 10 g of ground dried fruits are to be given orally twice daily with a cup of water.	Powder	7	4.86	0.05	0.09
*Carica papaya* L.	ببايا	Papaya	Caricaceae	Pharm-PCT-2761	Kidney stones	Seeds/ About 10 g of ground seeds are to be given orally twice daily.	Powder	5	3.47	0.03	0.06

In the case of kidney stones, the highest use values were for *Paronychia Argentina*, *Plantago ovata, Punica granatum, Taraxacum syriacum, Morus alba* and *Foeniculum vulgare*, respectively. In the case of urinary tract infections, the highest use values were for *Capsella bursa-pastoris, Ammi visnaga,* and *Ammi majus*, respectively. Besides, the maximum use value in case of renal failure was for *Portulaca oleracea* while the highest use values in the case of enuresis were for *Curcuma longa* and *Crocus sativus*, respectively. In the case of prostatic enlargement, the highest use values were for *Juglans regia, Quercus infectoria, Sambucus ebulus* and *Zea mays,* respectively. Furthermore, the frequencies of citation for these plants species were more than 50%.

The factor of informant’s consensus (F_ic_) was calculated for medicinal plants used for the treatment of various urinary tract diseases (i.e., 0.99 for Benign Prostate Hyperplasia (BPH) and enuresis and 0.98 in a case of kidney stones disease, urinary tract infection and renal failure). The calculated F_ic_ value obtained for the reported diseases indicated the degree of shared knowledge among informants for the treatment of these urinary tract diseases by certain medicinal plants as shown in Table 3.

**Table 3:** Factor of informant’s consensus (F_ic_) for the studied urinary tract diseases

Urinary tract disease categories	Nt	Nur	F_ic_
Kidney stones	22	1202	0.98
Urinary infection	17	912	0.98
Prostatic enlargement	8	544	0.99
Enuresis	7	496	0.99
Renal failure	10	370	0.98

Where; Nur is the number of use citations in urinary tract disease category, Nt is the number of taxa used for treatment of these diseases.

Ethnopharmacological information obtained from the study area on medicinal plants used in the treatment of various urinary tract diseases revealed that 57 plant species belonging to 30 families. All of the Latin scientific names have been checked with www.theplantlist.org on March 10, 2016.

**Fig. 2:**
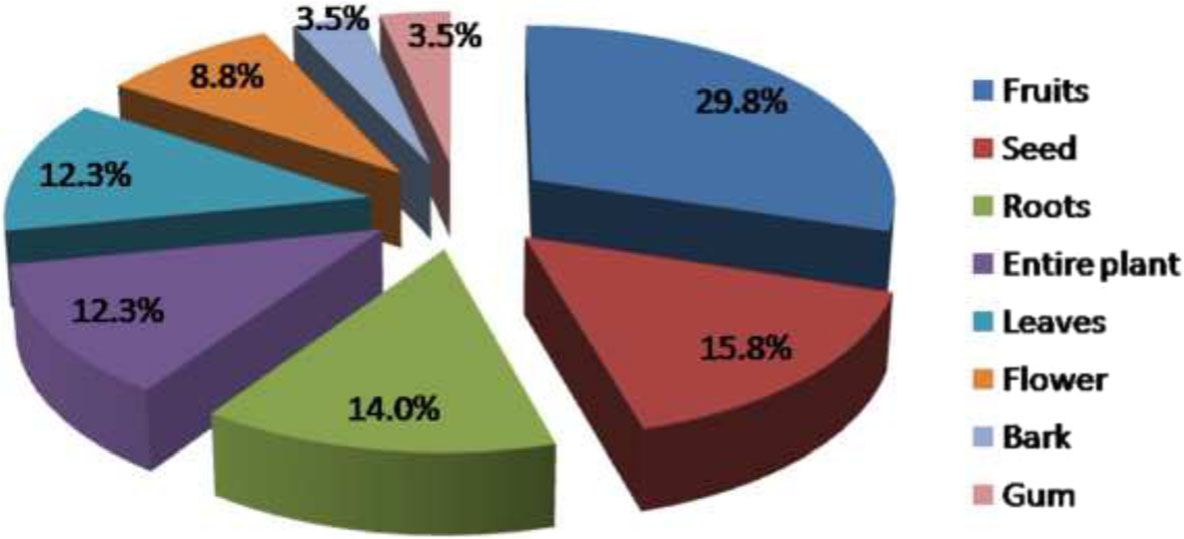
The frequency of the used parts of medicinal plants in the treatment of some urinary tract diseases

As presented in Table 4, the family use value was the highest for Apiaceae family, which was 26.67, where the most common plant parts used were fruits, seeds, and roots, respectively as shown in Fig. 2.

**Table 4:** Medicinal plant families used for treatment of urinary tract diseases and the family use value (30 families)

Number	Families	Number of taxa	Family use value
1.	Apiaceae	8	26.67
2.	Lamiaceae	5	16.67
3.	Leguminosae	5	16.67
4.	Brassicaceae	4	13.33
5.	Poaceae	3	10.00
6.	Rosaceae	3	10.00
7.	Compositae	2	6.67
8.	Cucurbitaceae	2	6.67
9.	Malvaceae	2	6.67
10.	Moraceae	2	6.67
11.	Solanaceae	2	6.67
12.	Other families with one citation	19	63.33

### Pharmaceutical preparations

**Fig. 3:**
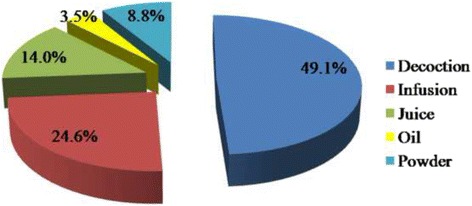
The frequency of preparation methods of medicinal plants for treatment of some urinary tract diseases

The methods of preparation were decoctions, infusions, juice, oil, and powder. Decoctions and infusions were the most frequently used methods of preparation as presented in Fig. 3.

**Fig. 4:**
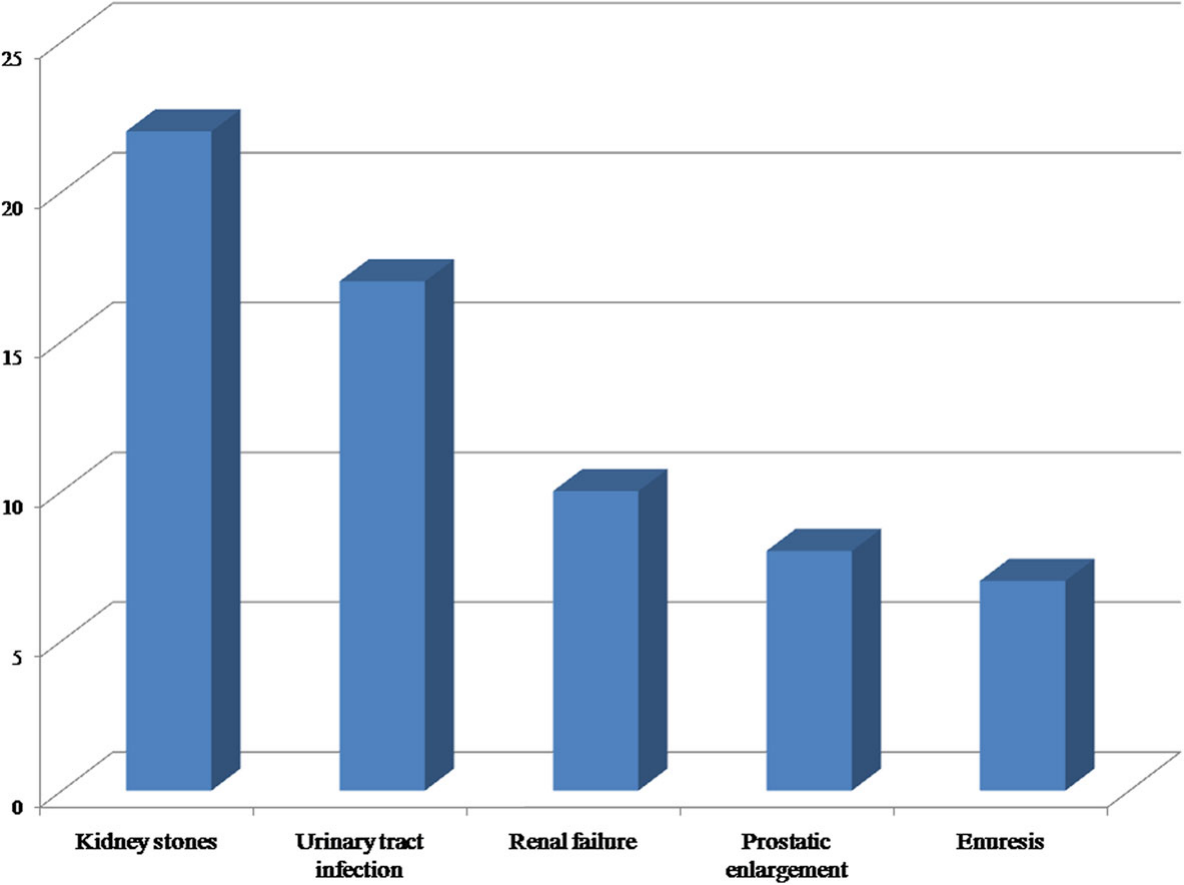
The frequency of urinary tract diseases mentioned by herbalists and traditional healers in the West Bank area of Palestine

The most common urinary tract disease treated with herbal remedies was kidney stones followed by urinary tract infections, renal failure, Benign Prostate Hyperplasia (BPH) and enuresis as reported in Fig. 4.

#### Literature review

For all the listed above plants, a literature review was investigated, where it represented their ethnopharmacological use against urinary tract diseases regionally, internationally and globally. Also, in-vitro as well as in-vivo studies and their toxic or adverse reactions were reviewed for plants which had F**C** value more than 50%, using electronic databases and the results were summarized in Table 5.

**Table 5:** Summary of published ethnopharmacology, in vivo, in vitro studies, side effects and toxicity of the most frequently recommended plants for treatment of urinary tract diseases

Urinary tract diseases	Plant species	Ethnopharmacological usage for treatment of various urinary tract diseases and country with reference source	In-vitro and in-vivo studies on plants used for treatment of various urinary tract diseases with reference source	Side effects and toxicity of plants used for treatment of various urinary tract diseases with reference source
Kidney stones	*Paronychia argentea*	Turkey [30] Jordan [26, 31, 32], Palestine [33, 34], Spain [35], Egypt [36], and Algeria [37].	In-vitro study on wistar rats it prevented and reduced the growth of kidney stones in experimental calcium oxalate nephrolithiasis [38].	In-vitro study proved that *P. argentea* extract had low hemolytic effect [39] as well as the butanolic extract of *P. argentea* can prevent or slow down the oxidative damage induced by organophosphorus pesticide, chloropyriphos ethyl in rats [38].
	Plantago ovata	No references found about its folk usage for treatment of kidney stones.	In vitro study on rats proved that intake of a *P. ovata* husk-supplemented diet prevented endothelial dysfunction [40].	Arabinoxylan from *Plantago ovata* husks had been proven its safety scientifically on rats and rabbits [41].
	*Punica granatum*	India and North Africa [42–45].	On male rats experiment proved that the administration of *P. granatum* induced urolithiatic rats resulted in removal of deposition of calcium oxalate crystals into kidneys and improving renal histology [46]. In another experiment on rats showed the protective effect of *p. granatum* in the ethylene glycol induced crystal depositions in kidneys [47].	No references
	*Taraxacum syriacum*	No references	No references	No references
	*Morus alba*	Bulgaria and Italy [48].	Ethanlic leaves extract showed significant anti-nephrolithiatic effect in wistar rats [49].	No reference
	*Foeniculum vulgare*	United Kingdom [50], Palestine [51], Italy [52], Turkey [53], Bosnia [54], Serbia [55], Iran [10, 56, 57], Pakistan [58], India [59], and Bolivia [60].	Herbal beverage of *F. vulgare* inhibited of calcium oxalate renal crystals formation in rats [61].	In most toxicity experiments carried out on *F. vulgare*, no signs of toxicity were observed [62].
Urinary tract infections	*Capsella bursa-pastoris*	In Jordan, [32] Turkey [63], Bulgaria [64], India [65], and Uzbekistan [66].	The crude extract of *C. bursa-pastoris* showed antibacterial activity against five Gram-positive and four Gram-negative bacteria. Among them *Escherichia coli* which is the mainly cause of urinary tract infections [67, 68].	*C. bursa-pastoris* extracts have been reported to exhibit low toxicity in mice, furthermore the plant is contraindicated in case of pregnancy [69], but used as an edible vegetable, eaten raw or cooked in some countries [70, 71].
	*Ammi visnaga*	Italy, Tunisia [72], Palestine [51], Lybia [73], Sudan [74], Egypt [75], Pakistan [76], and Peru [77].	*A. visnaga* has antifungal, antibacterial and antiviral activities due the presence of khellin and visnagin [78].	Overdose or longer use of *A. visnaga* can lead to queasiness, dizziness, loss of appetite, headache, sleep disorders and it should be avoided during pregnancy [79].
	*Ammi majus*	Italy, [80] Jordan [81] and Morocco [82].	The extract of *A. majus* has shown good inhibition in all the bacterial strains used specially *Escherichia coli* [83].	In vitro study on Geese showed severe liver damage in these birds which fed *A. majus* and exposed to sunlight [84].
Renal failure	*Portulaca oleracea*	India [85, 86] and Sri Lanka [87].	The ethanolic extract of the plant aerial parts showed significant anti-inflammatory and analgesic after intraperitoneal and topical applications but not oral administration when compared with the synthetic drug, diclofenac sodium as the active control [88]. Also its aqueous extract attenuates diabetic nephropathy through inhibition of renal fibrosis and inflammation in mice [89]. Although aqueous and ethanolic extracts of *P. oleracea* showed potential activity against cisplatin induced acute renal toxicity was studied in rats [90].	Due to the huge a mounts of oxalic acid and nitrate in the plant, a high consumption is harmful [91, 92].
Enuresis	*Curcuma longa*	No reference	No reference	No reference
	*Crocus sativus*	No references	No references	Histological studies indicated that saffron has not any toxic effect on liver [93], heart and spleen on mice and rats [94, 95].
Benign prostate hyperplasia (BPH)	*Juglans regia*	Palestine [96].	No reference	Juglone compound when isolated from all plant parts has multiple effects on cells such as the reduction of p53 protein levels, induction of DNA damage, inhibition of transcription and induction of cell death [97].
	*Quercus infectoria*	No reference	Contains quercetin which is at 500 mg 2 times daily gave significant symptomatic improvement to most patients, particularly those with negative expressed prostatic secretions cultures [98].	The aqueous extract of *Q. infectoria* has significant toxic effect in Wistar rats for over 180 consecutive days of consumption [99].
	*Sambucus ebulus*	Turkey [100], and Bosnia [101].	The plant extract produced significant inhibition of edema induced by carrageenan at all doses when compared to the control rats group [102].	The plant extract showed severe toxicity (in particular severe liver abscess) in all mice at all tested doses [102].
	*Zea mays*	Algeria, [103] Guatemala [104], Serbia, [105] Cameroon [106], Peru [107], Australia [108], Brazil [109], and Turkey [110].	Crude ethanolic extract of corn silk (stigma of *Zea mays*) exhibited a significant activity in anti-inflammatory herbal drugs for TNF (tumor factor-alpha) antagonistic activity. [111].	No adverse effects have been noticed with the consumption of corn silk which support the safety of corn silk for humans [112].

## Discussion

In the West Bank area of Palestine, the folk medicine has been trusted and highly appreciated, and many patients go to herbalists or traditional herbal healers to get benefit from this field. In fact, herbal medicine is considered the most used complementary and alternative medicine and this part of complementary and alternative medicines are widely used among patients suffering from urinary tract diseases throughout the world. Most practitioners are males, and this was confirmed in this study; some of them have university degrees.

All over the world, the prevalence of kidney diseases varies significantly from country to country. Epidemiological data on the occurrence of kidney stone was about 12% of global population with a recurrence rate of 70–80% in males and 47–60% in females [29].

According to the use value results, the highest use values for medicinal plants, which were utilized for the treatment of kidney stones, were for *Paronychia argentea*, *Plantago ovata*, *Punica granatum*, *Taraxacum syriacum*, *Morus alba, and Foeniculum vulgare*. The highest use values for medicinal plants used for treatment of urinary tract infections were for *Capsella bursa-pastoris, Ammi visnaga,* and *Ammi majus*, while the highest use value for plants used for treatment of renal failure was for *Portulaca oleracea* as well as the highest use values for medicinal plants used for treatment of enuresis were for *Curcuma longa* and *Crocus sativus*. Furthermore the maximum use values for plants used for treatment of benign prostate hyperplasia were for *Juglans regia, Quercus infectoria, Sambucus ebulus* and *Zea mays.*

Table 5 showed that *Paronychia argentea*, *Punica granatum*, *Morus alba*, and *Foeniculum vulgare* were used in the folk medicine in many countries for the treatment of kidney stones. The evidence-based effects for this disease were documented for *Paronychia argentea*, *Plantago ovata*, *Punica granatum*, *Morus alba*, and *Foeniculum vulgare*, whereas *Taraxacum syriacum* lacked any evidence-based use for treatment of kidney stone. Moreover, specific attention must be considered during consumption of *Paronychia argentea* extract, which has a low hemolytic effect.

Regarding the most cited plants which were used for the treatment of urinary tract infections, all of them applied in the folk medicine in many countries and their antibacterial effect approved scientifically, but all of them may have harmful effects due to their adverse reactions and toxicological effect. Mean while the most cited plant for treatment of renal failure was *Portulaca oleracea* which is used in India and Sri Lanka for the treatment of this disease also, but unfortunately, this plant contains oxalic acid and nitrate. Therefore, high consumption of this plant is harmful to patients suffering from renal failure. Moreover, the most cited plants for treatment of enuresis were *Curcuma longa* and *Crocus sativus* which was not mentioned before in any folk or evidence-based medicines for the treatment of this disease and their toxicological effects were not reported.

The most cited plants which were used for the treatment of benign prostate hyperplasia (prostatic enlargement) were; *Juglans regia*, *Sambucus ebulus* and *Zea mays*. These plants had evidence-based studies to be useful for the treatment of this disease, but it is important to keep in mind that *Juglans regia*, *Quercus infectoria* and, *Sambucus ebulus* are harmful as mentioned in the literature review and cannot be used for treatment of any disease.

Over all, there are quite a few phytopharmaceuticals which can be used effectively for the treatment of the urinary tract diseases in the pharmaceutical markets. For that further phytochemical and pharmacological screenings is required to investigate new drugs from the mentioned plants in this study, especially those which have high use values and can be used safely.

## Conclusion

The traditional herbal medicine has gradually become more popular, and the need for promoting awareness is perceived. This study showed that traditional herbal medicine is playing a significant role for treatment of urological diseases in the West Bank of Palestine. Based on that, all the plants in this study with high use value should have further phytochemical and pharmacological screenings to test for safety and efficacy. Despite the fact that many of the herbals are currently used by local and international herbalists and traditional healers, serious attention must be given toward many of these products, since they have serious adverse effects and toxicities. *Curcuma longa* and *Crocus sativus* were the most cited plants for treatment of enuresis. These plants could be of interest for additional research since they have not been mentioned before in any folk or evidence-based medicines for the treatment of this disease and their toxicological effects were not reported. Also, it is important to keep in mind that *Juglans regia, Quercus infectoria* and, Sambucus ebulus are harmful and cannot be used for the treatment of any disease.

## Abbreviations

BPH: Benign Prostate Hyperplasia
CV: Choice Value
FC: frequency of citation
F_ic_: Factor of Informant’s Consensus
IRB: Institutional Review Board
ns: Total Number of Species
Nt: Number of Taxa
Nur: is the number of use
UV: Use Value
*UVs*: Use Values of the Species
